# Usefulness of DWI in preoperative assessment of deep myometrial invasion in patients with endometrial carcinoma: a systematic review and meta-analysis

**DOI:** 10.1186/s40644-014-0032-y

**Published:** 2014-11-12

**Authors:** Sushant K Das, Xiang K Niu, Jing L Wang, Li C Zeng, Wen X Wang, Anup Bhetuwal, Han F Yang

**Affiliations:** 1 Department of Radiology, Affiliated Hospital of North Sichuan Medical College, 63 Wenhua Road, Nanchong 637000, Sichuan, People’s Republic of China; 2 Department of Radiology, Affiliated Hospital of North Sichuan Medical College, 63 Wenhua Road, Nanchong 637000, Sichuan, People’s Republic of China

**Keywords:** Diffusion-weighted imaging, Magnetic resonance imaging, Endometrial carcinoma, Myometrial invasion

## Abstract

**Background:**

The objective of this study was to perform a systematic review and a meta-analysis in order to estimate the diagnostic accuracy of diffusion weighted imaging (DWI) in the preoperative assessment of deep myometrial invasion in patients with endometrial carcinoma.

**Methods:**

Studies evaluating DWI for the detection of deep myometrial invasion in patients with endometrial carcinoma were systematically searched for in the MEDLINE, EMBASE, and Cochrane Library from January 1995 to January 2014. Methodologic quality was assessed by using the Quality Assessment of Diagnostic Accuracy Studies tool. Bivariate random-effects meta-analytic methods were used to obtain pooled estimates of sensitivity, specificity, diagnostic odds ratio (DOR) and receiver operating characteristic (ROC) curves. The study also evaluated the clinical utility of DWI in preoperative assessment of deep myometrial invasion.

**Results:**

Seven studies enrolling a total of 320 individuals met the study inclusion criteria. The summary area under the ROC curve was 0.91. There was no evidence of publication bias (P = 0.90, bias coefficient analysis). Sensitivity and specificity of DWI for detection of deep myometrial invasion across all studies were 0.90 and 0.89, respectively. Positive and negative likelihood ratios with DWI were 8 and 0.11 respectively. In patients with high pre-test probabilities, DWI enabled confirmation of deep myometrial invasion; in patients with low pre-test probabilities, DWI enabled exclusion of deep myometrial invasion. The worst case scenario (pre-test probability, 50%) post-test probabilities were 89% and 10% for positive and negative DWI results, respectively.

**Conclusion:**

DWI has high sensitivity and specificity for detecting deep myometrial invasion and more importantly can reliably rule out deep myometrial invasion. Therefore, it would be worthwhile to add a DWI sequence to the standard MRI protocols in preoperative evaluation of endometrial cancer in order to detect deep myometrial invasion, which along with other poor prognostic factors like age, tumor grade, and LVSI would be useful in stratifying high risk groups thereby helping in the tailoring of surgical approach in patient with low risk of endometrial carcinoma.

## Background

Endometrial Cancer is the seventh most common malignant disorder, the most common female gynecologic cancer, and the fourth most common cancer in women [1]. Histopathological markers for recurrence and poor outcomes in endometrial cancer are becoming increasingly important in treatment planning. The PORTEC-1 trial [2] established certain factors for recurrence in early stage patients including age, high grade histology, and depth of myometrial tumor invasion, which was further validated by Gynecologic Oncology Group (GOG) study 99 [3] into a subgroup of patients who were deemed as “high intermediate risk” (HIR) for recurrence and decreased overall survival based on age, tumor grade, lymph vascular space invasion (LVSI), and depth of myometrial invasion [4]. The depth of myometrial invasion is defined as superficial i.e. confined to endometrium or inner half (<50%) of the myometrium; and deep invasion, i.e., invading the outer half (≥50%) of the myometrium, in contrast to historical definition where it was defined as superficial, i.e., outer 1/3rd (33%), middle 1/3rd (<66%) and inner 1/3rd (≥66%) of myometrial invasion [4],[5]. These variables (age, tumor grade, LVSI and depth of myometrial invasion) display strong inter-relationship since G1 and G2 tumors tend to be less invasive and to have less LVSI, while G3 tumors are more often deeply invasive, and have a greater frequency of LVSI. It is not clear if there is an independent prognostic variable [4],[6]. In particular, histological tumor grade and depth of myometrial invasion strongly correlate with the presence of lymph node metastases and overall patient survival [7]. The incidence of lymph node metastases increases from 3% with superficial myometrial invasion to 46% with deep myometrial invasion [8],[9]. Although, more recently, several studies showed the LVSI as a much stronger independent prognostic factor for lymph node metastasis, but this does not imply that outer-third invasion and Grade 3 histology are not important poor prognostic factors. Zhang et al. [6], in their study, found that patients with age greater than 60 years and positive LVSI were associated with increased local regional recurrence, while progesterone receptor (PR) status and the depth of myometrial invasion had significant impacts on distant failure. Therefore, preoperative information about the depth of myometrial invasion is essential in tailoring the surgical approach for these patients. The myometrial invasion ratio determines the International Federation of Gynecology and Obstetrics stage and thus has a direct influence on treatment. Although clinical guidelines of the National Cancer Center Network from the United States require a complete dissection of pelvic and para-aortic nodes regardless of the estimated myometrial invasion [10], certain European groups advocate a less aggressive surgical approach, with surgical lymphadenectomy used only for patients with deep myometrial invasion [1]. Furthermore, clinical judgment of absent myometrial involvement is critical for young patients with Grade 1 endometrial adenocarcinoma since fertility-preserving treatment may be an option [11].

To date, magnetic resonance imaging (MRI) is an accurate imaging technique for preoperative assessment of endometrial cancer and for evaluating the depth of myometrial invasion [12]-[14]. A recent meta-analysis demonstrated that contrast enhanced T1-weighted (T1WI) MRI was substantially better than ultrasonography, CT, or noncontrast MRI [15]. Moreover, dynamic contrast enhanced MRI (DCE-MRI) is considered more accurate than T2-weighted imaging (T2WI) in tumor detection and in assessing myometrial invasion due to greater contrast and clearer demonstration of the border between the tumor and myometrium in the early phase [16]-[18]. However, recent concerns with respect to the development of nephrogenic systemic fibrosis in patients with renal insufficiency, who undergo contrast enhanced MRI [19], are increasing the need of non-enhanced imaging modalities that might be useful for preoperatively evaluating endometrial cancer. A few studies reported that DWI might be useful for detecting the depth of myometrial invasion with high diagnostic accuracy [20] as well as predict tumor grade [21]. The objective of this study was to assess the overall diagnostic value of DWI in the preoperative assessment of deep myometrial invasion in patients with endometrial cancer with a meta-analysis, which to our knowledge had not previously been studied.

## Methods

Published methods were used to identify relevant studies, assess study eligibility, evaluate the methodologic quality of the studies [22]-[24], and summarize the diagnostic accuracy findings [22]-[25].

### Search strategy

A comprehensive computer literature search was performed to identify English articles examining the diagnostic accuracy of Diffusion Weighted-Magnetic Resonance Imaging (DW-MRI alone, DWI combined with T2 or DWI with fused T2 images) for detection of deep myometrial invasion in patient with endometrial cancer. The MEDLINE, EMBASE databases, and Cochrane Library were searched from January 1995 to January 2014. For the electronic search, a search algorithm was used that was based on a combination of the following keywords: (“Diffusion-weighted magnetic resonance images” OR “Diffusion Magnetic Resonance” OR “DW-MRI” OR “DW magnetic resonance images”) AND (“endometrial Neoplasm OR endometrial carcinoma” OR “myometrial invasion”) AND (sensitivity OR specificity OR false negative OR false positive OR diagnosis OR detection OR accuracy). Other databases such as Cancerlit and Cochrane Library were also searched for relevant articles. The reference lists of included studies and review articles were manually searched.

### Study selection

Two investigators, Xiang Ke Niu and Jing Liang Wang, who were blinded to the journal, author, institution, and date of publication, independently checked retrieved articles. According to a standardized data extraction form, all the abstracts were read to identify the potentially eligible articles, and after retrieving the full text of these articles it was determined whether they were exactly eligible or not. The inclusion criteria were as follows: (a) Articles were published in English; (b) DW-MRI (DWI without referring to T2 images, T2 + DWI and DWI with fused T2 images) was used to evaluate myometrial invasion in endometrial cancer patient; (c) Uniform standard of reference: depth of myometrial invasion assessed with histologic specimen from the uterus obtained at surgery; (d) Results were reported in sufficient detail to reconstruct contingency tables of the raw data (i.e., true-positive, true-negative, false-positive, and false-negative findings). The authors of studies not reporting with sufficient data were contacted to request for additional information; (e) Concerning the quality of study design, only articles in which the number of “yes” answers to the 14 questions in the Quality Assessment of Diagnostic Accuracy Studies (QUADAS) tool [26] was more than nine were included.

### Data extraction and quality assessment

The same two investigators who performed the database searches also performed the relevant data extraction independently. In order to resolve any disagreement between the reviewers, a third reviewer, Li Chuan Zeng, assessed all points of disagreement. The majority opinion then was used for analysis. Relevant studies were further examined with the QUADAS criteria again. To perform accuracy analyses, data was extracted on the characteristics of studies and patients; measurements performed and results. For each report, the following data items were extracted: author; year of publication; sample size; description of study population (age); study design; patient enrollment; inclusion and exclusion criteria, reasons for exclusions from the analysis; DW-MRI test methods; number of lesions and interpretation of the test results (blinded or not).

For each study, 2 × 2 contingency tables were extracted or reconstructed. If diagnostic accuracy was compared between different groups of observers, only one contingency table for the findings by observer with highest experience was included.

### Data synthesis and statistical analyses

The primary outcome in the analyses was the performance of DWI in the diagnosis of deep myometrial invasion in patients with endometrial cancer, as quantified in terms of sensitivity, specificity, diagnostic log odds ratios and likelihood ratios with corresponding 95% confidence intervals.

### Meta-analytical model

A bivariate mixed-effects regression model [27],[28] was used to summarize the diagnostic results of this study. This bivariate approach accounts for potential between-study heterogeneity and incorporates the possible correlation between the sensitivity (SEN) and the specificity (SPE) [28].

### Summary performance estimates

Summary sensitivity and specificity were calculated by using a bivariate regression approach and derived corresponding positive likelihood, negative likelihood and diagnostic odds ratios as functions of these summary estimates. The derived estimates of sensitivity, specificity, and respective variances were also used to construct a summary ROC curve [29],[30]. The area under the ROC curve was used as an alternative global measure of test performance [31]. The DOR which is a single indicator of test accuracy that comprises a combination of sensitivity and specificity information was also calculated [32]. The post-test probability of deep myometrial invasion (Ppost) was calculated from likelihood ratios by using the Bayes theorem [33].

Positive and negative likelihood ratios (LRs) characterize the clinical utility of a test and are used to estimate the post-test probability of disease. The likelihood ratios were used to simulate three clinical scenarios by using different pre-test probabilities of deep myometrial invasion (25%, indicating low clinical suspicion (grade 1); 75%, indicating high clinical suspicion (grade 3); and 50%, indicating worst case scenario (grade 2), and by using the likelihood ratios, post-test probabilities were calculated and plotted on Fagan nomograms. Likelihood ratios higher than 10 and lower than 0.1 indicate that the given test generate strong evidence to rule in or rule out a diagnosis, respectively.

Forest plots, with statistical assessments performed by using the ×^2^ test of homogeneity and the inconsistency index (I^2^) was used to assess between study variation (Heterogeneity) where an inconsistency index of 0% indicates no observed heterogeneity, and values greater than 50% is considered to indicate substantial heterogeneity [34].

In test accuracy studies, one of the primary causes of heterogeneity is the threshold effect, which arises when different cut-offs or thresholds are used in different studies to define a positive (or negative) test result. The Spearman correlation coefficient between the logit of sensitivity and the logit of (1-specificity) was computed to assess the threshold effect. A strong positive correlation would suggest a threshold effect, P <0.05 [35],[36]. Also the SROC curve illustrates the relationship between SEN and SPE, determining the presence of a threshold effect where a typical pattern “shoulder arm” plot in a ROC space is thought to suggest threshold effect [36],[37]. A perfect test has a summary ROC curve with an area close to 1.0 whereas poor tests have an area under the ROC curve close to 0.5.

Publication bias was assessed visually by using a scatter plot of the inverse of the square root of the effective sample size (1/√ESS ) versus the diagnostic log odds ratio, which would have a symmetric funnel shape when publication bias was absent. Formal testing for publication bias was conducted by using a regression of the diagnostic log odds ratio against 1/√ESS and weighting it according to the effective sample size, with P <0.10 indicating significant asymmetry [38].

The data was analyzed by using the MIDAS (Meta-analytical Integration of Diagnostic Accuracy Studies) command in the Stata 12.0 software (Stata Corporation, College Station, TX, USA) except threshold effect, which was analyzed by using Metadisc version 1.4 (Ramón y Cajal Hospital. Madrid, Spain).

## Results

### Literature search and selection of studies

**Figure 1:**
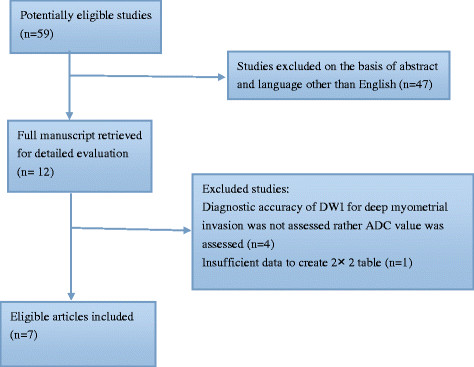
Flow diagram of studies identified in the systematic review.

The detailed procedure of study selection in the meta-analysis is shown in Figure 1. An electronic search yielded 59 primary studies of which 47 non-pertinent articles were excluded after reviewing the title and abstract. Then full-text reports were obtained for 12 publications. Five articles were excluded after reviewing the full article for the following reasons: (a) the aim of the articles was not to reveal the diagnostic value of DWI for assessment of deep myometrial invasion in patient with endometrial cancer [39]-[42], and (b) researchers in the articles did not have enough data that could be used to construct or calculate true-positive, false-positive, true-negative and false-negative results [43]. A total of seven studies [5],[20],[44]-[48], which fulfilled all of the inclusion criteria were considered for the analysis.

### Study characteristics

The seven evaluated studies involved 320 women diagnosed with endometrial cancer. Of those diagnosed, 79 of them had deep myometrial invasion while 241 had superficial or no invasion on histopathological examination. The trials were published between 2000 and 2013. Five trials were prospective and enrolled patient consecutively, while others were retrospective and did not enroll patient consecutively or the manner of enrollment was not specified. All of the studies used DWI alone as an index test to determine deep myometrial invasion. A histopathological test was used as a reference gold standard test in each study. Table 1 shows the principal characteristics of the 12 studies included in the meta-analysis.

**Table 1:** Description of studies included in meta-analysis

**Author**	**Year**	**Journal**	Study patient**	**Total No. of patient**	**Age (range)**	**Index test**	**Field strength**	**b-value (s/mm**^**2**^**)**	**Time interval**
Shen	2008	AJR	Pro	21	33 - 82 years	DWI + T2	1.5T	1,000	NR
Takeuchi	2009	Acta Radiol	Retro	33	24 - 85 years	DWI + T2	*1.5 or 3.0T	0 and 800	NR
Lin	2009	Radiology	Pro	48	25-80 years	DWI with Fused T2	3.0T	0 and 1000	1 - 21 days
Rechichi	2010	Eur Radiol	Pro	47	36 - 84 years	Only DWI (no referring to T2)	1.5T	0 and 500	5-35 days
Beddy	2012	Radiology	Retro	48	NR	DWI + T2	1.5T	0 and 800	NR
Seo		J Magn Reson Imaging	Pro	52	29 - 75 years	DWIBS with fused T2	3.0T	1000	1 - 20 days
Hori	2013	Eur Radiol	Pro	71	31 - 82 years	DWI + T2 Also referred to DWI with Fused T2	3.0T	0 and 1000	Less than 70 days

Note:

The study identification (ID) numbers correspond to study numbers on the graphs in Figures 5.

Time interval is the time between DWI examination and surgical procedure to histopathologically confirm the extent of myometrial invasion.

All studies had a standard reference of histopathology i.e., all the patients in each study had pathological confirmation of endometrial cancer by Dilation and Curettage or endometrial biopsy.

All studies enrolled patients consecutively.

NR = not reported.

*Some patients were evaluated using Magnetic field strength 1.5T while others with 3.0T.

**Pro = Prospective, Retro = Retrospective.

### Assessment of study quality and publication bias

**Figure 2:**
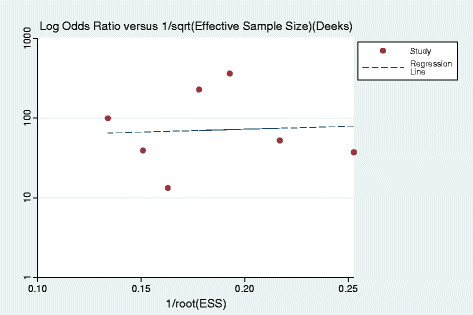
**Results of Deek’s funnel plot asymmetry test for publication bias.** The non-significant slope indicates that no significant bias was found. ESS = effective sample size.

All seven studies included in the meta-analysis fulfilled nine or more of the 14 criteria in the Quality Assessment of Diagnostic Accuracy Studies tool for methodologic quality. The results of this assessment are presented in Table 2. Results of the funnel plot asymmetry test for publication bias analyzed by means of linear regression of the log odds ratio on effective sample size were not significant (bias coefficient, 1.69; P = 0.90), and the slope was not significant, which suggested no major publication bias (Figure 2).

**Table 2:** Criteria found and not found in selected studies according to quality assessment of diagnostic accuracy studies tool

**Criterion**	**Shen et al.**	**Takeuchi et al.**	**Lin et al.**	**Rechichi et al.**	**Beddy et al.**	**Seo et al.**	**Hori et al.**
Patient spectrum	Yes	Yes	Yes	Yes	Yes	Yes	Yes
Selection criteria	Yes	Yes	Yes	Yes	Yes	Yes	Yes
Reference standard	Yes	Yes	Yes	Yes	Yes	Yes	Yes
Time between test	Unclear	Unclear	Yes	Yes	Unclear	Yes	Yes
Partial verification	Yes	Yes	Yes	Yes	Yes	Yes	Yes
Differential verification	Yes	Yes	Yes	Yes	Yes	Yes	Yes
Incorporation	Yes	Yes	Yes	Yes	Yes	Yes	Yes
Index test, sufficient detail	Yes	Yes	Yes	Yes	Yes	Yes	Yes
Reference test, sufficient detail	Yes	Yes	Yes	Yes	Yes	Yes	Yes
Test bias	Yes	Yes	Yes	Yes	Yes	Yes	Yes
Review bias	Unclear	Unclear	Unclear	Unclear	Unclear	Unclear	Unclear
Clinical data	Unclear	Yes	Yes	Yes	Yes	Yes	Yes
Un-interpretable data	Unclear	Unclear	Unclear	Unclear	Unclear	Unclear	Unclear
Subject withdrawal	Yes	Yes	Yes	Yes	Yes	Yes	Yes

Note: yes = criterion found in given study, No = criterion not found in given study, Unclear = Insufficient information to make judgment.

### Diagnostic accuracy

**Figure 3:**
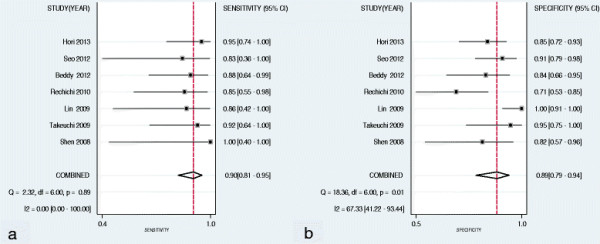
**Forest plots of studies involving evaluation of the sensitivity and specificity of DWI in the diagnosis of deep myometrial invasion in patient with endometrial cancer.** Summary sensitivity **(a)** and specificity **(b)** of DWI for the diagnosis of deep myometrial invasion were 0.90 (95% CI: 0.81, 0.95) and 0.89 (95% CI: 0.80, 0.94), respectively.

The sensitivity and specificity of DWI for the diagnosis of deep myometrial invasion were 0.90 (95% CI: 0.81 - 0.95) and 0.89 (95% CI: 0.79 - 0.94), respectively (Table 3). Forest plots of the sensitivity and specificity of DWI in the diagnosis of deep myometrial invasion are shown in Figure 3. The likelihood ratio syntheses gave an overall PLR of 8.08 (4.21 - 15.49) and a NLR of 0.11 (0.06 - 0.22). The diagnostic odds ratio was 71.1 (95% CI: 24.96 - 202.29) (Table 3).

**Table 3:** Meta-analysis summary statistics

**Parameter**	**Estimates**
Sensitivity	0.90 [ 0.81 - 0.95]
Specificity	0.89 [ 0.79 - 0.94]
Positive LR	8.08 [ 4.21 - 15.49]
Negative LR	0.11 [ 0.06 - 0.22]
Diagnostic score	4.26 [ 3.21 - 5.31]
DOR	71.1 [ 24.96 - 202.29]

Note: Data are summary estimates for all of the studies with all readers. Numbers in parentheses are 95% Cls. DOR = diagnostic odds ratio, LR = likelihood ratio.

### Evaluation of clinical utility

**Figure 4:**
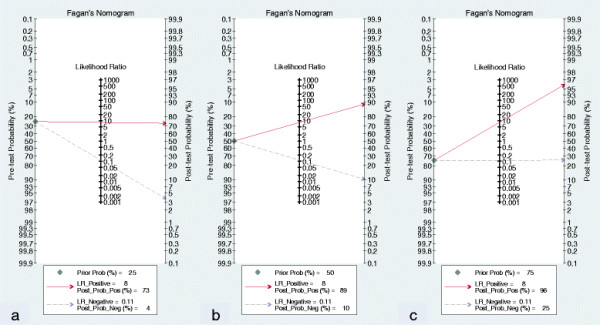
**Pretest probabilities (Prob) and likelihood ratios (LR). (a)** With a pretest probability of deep myometrial invasion of 25% (low clinical suspicion), the posttest probability of deep myometrial invasion, given a negative DWI result (Post-Neg Probability), is 4%, which can be considered sufficient to rule out deep myometrial invasion. **(b)** With a pretest probability of deep myometrial invasion of 50% (worst-case scenario), the posttest probabilities of deep myometrial invasion, given positive and negative DWI results, are 89% and 10%, respectively. Thus, it is a useful test in this situation. **(c)** With a pretest probability of deep myometrial invasion of 75% (high clinical suspicion), the posttest probability of deep myometrial invasion, given a positive DWI result (Post-Pos Probability), is 96%; thus, a positive DWI result can be considered sufficient to rule in deep myometrial invasion.

The positive and negative likelihood ratios of DWI for the diagnosis of deep myometrial invasion were 8.07 (95% CI: 4.21 - 15.48) and 0.11 (95% CI: 0.05 - 0.22), respectively. With a pre-test probability of deep myometrial invasion of 25% (low clinical suspicion), the post-test probability of deep myometrial invasion, given a negative DWI result, was 4%, which could be considered sufficient to rule out deep myometrial invasion (Figure 4a). With a pre-test probability of deep myometrial invasion of 75% (high clinical suspicion), the post-test probability of deep myometrial invasion, given a positive DWI result, was 96%. Thus, a positive DWI result could be considered sufficient to rule in deep myometrial invasion (Figure 4c). With a pre-test probability of DWI of 50% (worst-case scenario), the post-test probability of deep myometrial invasion, given a positive DWI result, was 89%, and the post-test probability of deep myometrial invasion, given a negative DWI result, was 10% (Figure 4b). Thus, DWI is a useful test in this situation.

### Heterogeneity and threshold effect

The inconsistency index for the overall heterogeneity of the study was 0% (95% CI: 0 – 100). It is noteworthy that I^2^ = 0% in our study should not be interpreted as homogeneous as I^2^ estimates may be unreliable due to lack of power and precision, due to the presence of time-dependent biases, or due to dependence on trial weights and precision [49]. In this study, there were only 320 events and 7 trials. It is believed that considerable fluctuations is likely to occur in I^2^ estimates when a meta-analysis includes less than roughly 500 events and less than 15 trials [49]. Moreover, if a meta-analysis is conducted at a time where all trials yield large promising treatment effects, the similarity across trials will result in a relatively small I^2^ estimate. In this study, all the included studies had higher diagnostic accuracy for DWI except for Rechichi and Shen [20],[46]. Also from the mathematical expression, I^2^ = τ^2^/ (σ^2^ + τ^2^), it is clear that relatively large sampling errors across trials will result in small I^2^ estimates [49].

**Figure 5:**
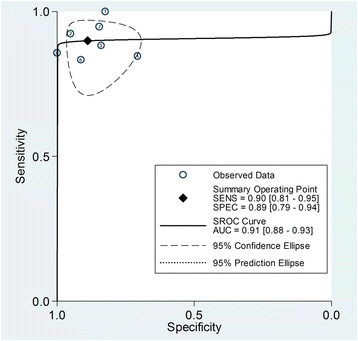
**Summary ROC (SROC) curve for the six included studies.** Numbers in brackets are 95% CIs. AUC = area under ROC curve, SENS = sensitivity, SPEC = specificity.

A Spearman rank correlation was performed as a further test for the threshold effect and was determined to be 0.214 (P = 0.645), which indicated that there was an absence of a notable threshold effect in the accuracy estimates among individual studies. Moreover, the pattern of the SROC curve was not of a “shoulder-arm” shape and the area under the ROC curve was close to 1.0 (Figure 5). A subgroup analyses was not performed in this current study owing to its relatively small study group.

## Discussion

The histologic tumor grade and the depth of myometrial invasion in patients with endometrial cancer correlate strongly with the prevalence of lymph node metastasis and with patient survival [50]. The reported five-year survival rate in patients with deep myometrial invasion or with grade 3 endometrial cancer is 58% to 59% compared with 89% for patients with Grade 1 endometrial cancer [8],[51],[52]. If there is a deep (>50%) myometrial infiltration, pelvic and lumbo-aortic nodal dissection must be added to hysterectomy and salpingoophorectomy [53]. Although some studies (ASTEC, Benedetti-Panici) [54],[55] showed no therapeutic benefit of combined pelvic and para-aortic lymphadenectomy for patients with endometrial cancer, these studies had some limitations. While in the ASTEC study, the follow-up period was short, lymphadenectomy was selective rather than systematic (nine or fewer lymph nodes were removed in 35% of patients in the lymphadenectomy group), neither of the studies included para-aortic lymphadenectomy [56],[57], which is an important site of endometrial carcinoma metastases as confirmed by sentinel lymph node investigation [58], and subsequent radiation was not tailored to the nodal status. Hence, it is difficult to draw any definite conclusion from these studies. Furthermore, findings from the SEPAL study [59] suggested that the survival effect of lymphadenectomy was restricted in low risk patients, but in patients with intermediate or high risk, a complete systematic lymphadenectomy in pelvic and para-aortic regions had substantial therapeutic effects. The authors recognize that, MR findings are an important predictor of lymph node metastases, which in combination with tumor grade and histologic assessment could be useful in identifying patients at low risk for recurrence. This being said, it could also not be denied that diagnostic imaging and tumor markers have not yet proved to be accurate enough in the evaluation of tumor extent to replace surgical staging although it may enable an optimization of the surgical procedure and result in a better tailored therapeutic strategy.

The diagnostic accuracy of MRI, CT, and US in assessing myometrial invasion has been extensively evaluated. Results of a recent meta-analysis [15] showed that, in the preoperative assessment of myometrial invasion, contrast material enhanced MRI in the pelvis was substantially better than US, CT, and nonenhanced MRI. However, its invasiveness and contraindication in patients with renal insufficiency increases the need of non-invasive test to evaluate myometrial invasion. DW-MRI offers potential advantages over DCE-MRI because it does not involve intravenous administration of a contrast agent and entails a shorter imaging time.

DWI is a recent prevailing technique that enables distinction between cancerous and normal tissues, determines lesion aggressiveness, and monitors responses to therapy by providing information on extracellular-space tortuosity and tissue cellularity [60]. The use of a high b value makes images more sensitive to water diffusion; hence, it increases contrast enhancement between normal and cancerous tissues, but with a drawback of decreased signal intensity and anatomic detail of the adjacent structures [61]. Thus referring to conventional T2 weighted image for an anatomical landmark is warranted. More recently, to overcome this morphologic deterioration on DWI, fusion of T2-weighted and high-b-value DWI has been applied for malignant tumor screening at 1.5 T [61] or 3T with improved signal to noise ratio [62]. Furthermore, Gallego et al. [63] found apparent diffusion coefficient (ADC) mapping overcomes the drawback of high b values, which was found to cause other lesion like hyperplasia also to appear hyper-intense on DWI. In addition to these refinements, a new DWI technique DWIBS (diffusion-weighted whole-body imaging with background body signal suppression) has been introduced which is considered useful in the organs that require the acquisition of images with free breathing; especially in the abdomen and pelvis [64],[65]. Moreover, DWI can also provide ADC values of tissue, which is considered to be influenced by nuclear-to-cytoplasm ratio (NCR) and cellular density [66]-[70]. Several studies have showed ADC measurement has a potential ability to differentiate between normal and cancerous tissue in the endometrium [71]-[73] and also suggested that ADC values of endometrial cancer of higher grade showed a tendency to decrease as compared with those of lower grade [73], but the mean ADC in assessing depth of myometrial invasion was found to be insignificant [40],[41],[43]. Although, Inoue et al. and Nakamura et al. [39],[42] suggested that a minimum ADC correlates with prognostic parameters of endometrial carcinoma more strongly than a mean ADC. A lower minimum ADC suggests higher histological tumor grade and deep myometrial invasion although more studies would be required to validate ADC measurement for assessing depth of myometrial invasion. However, multiple studies have shown DWI combined with T2 or with application of fused image to be effective in assessing deep myometrial invasion. So far myometrial invasion is usually determined by means of an intraoperative frozen section histological study. It is noteworthy to mention here that in the study by Gallego et al. [63], they found a statistically insignificant difference between frozen section histology and DWI in detecting myometrial invasion. In fact, sensitivity and NPV of the DWI sequence were slightly higher, which improves the possibilities of correctly identifying patients in early stages of the disease, in which there is no deep invasion, which is the main objective of pre- and intra-operative evaluations. Also, while along with deep myometrial invasion, tumor size as well as lymph node metastasis (LNM) can be assessed with MRI, it is limited in its capacity in detecting LNM, which led some authors to consider its role superfluous [74] or simply suggesting it should not be performed [75]. Morphologic imaging techniques for diagnosing nodal involvement (size of short-axis node diameter greater than 10 mm), have low sensitivity ranging from about 20% to 65%, with specificity between 73% and 99% [76]. Nonetheless, in the study of Lin et al. [45], the criteria of combining lymph node size and relative ADC value increased the sensitivity from 25% to 83% for MRI in the detection of LNM in endometrial carcinoma. However, the objective of the present study was to evaluate the diagnostic accuracy of DWI in assessing deep myometrial invasion in patients with endometrial carcinoma.

The meta-analysis results reported herein showed that DWI has excellent accuracy in the diagnosis of deep myometrial invasion, with an area under the ROC curve of 0.91. The pooled PLR of 8 suggest that patients with deep myometrial invasion have approximately 8 fold chance of having DWI positive compared to patients with superficial or no myometrial invasion. On the other hand, the pooled NLR of 0.1 suggest that if the DWI was negative, the probability that this patient has deep myometrial invasion was 0.1%, which is low enough to rule out deep myometrial invasion.

### Limitations

Some limitations should be considered when interpreting the results. First, the sample sizes of several included studies were rather small and they may not have had adequate ability to assess the diagnostic accuracy. Second, not all the studies used similar MRI protocols in the assessment of deep myometrial invasion. There were differences in DWI parameters, namely: magnet field strength (1.5T vs. 3T), type of coil and b-value across the studies, which could not be compared here, owing to relatively small number of publication that met the inclusion criteria. Rechichi et al. [20] used DW-MRI without referring to T2 weighted imaging while several other studies either referred [5],[44],[46]-[48] or solely [45] used fusion images for the assessment of deep myometrial invasion. It is noteworthy that fusion images are superior to solo DWI or even to DWI combined conventional T2 images and thus the latter two might result in misdiagnosis and cause more false negative results. Also diffusion weighted imaging with background suppression (DWIBS) used by Seo et al. [44] is considered to have better resolution as compared to DWI without background suppression. Furthermore, he fused DWIBS onto T2 images that might have caused better depiction of deep myometrial invasion in his study. Moreover, not all studies had similar planar reconstruction images. Hori et al. [48] suggested that overall quality regarding the ability to assess tumor extent was better with 3D-TSE imaging than 2D-TSE imaging. Apart from all the variability between studies, different magnetic fields were also used within one study done by Takeuchi et al. [47] where different field strengths might have affected diagnostic results as some patients were evaluated with 1.5T while others with 3.0 T. Finally, this meta-analysis was limited to published (English) studies that may have negated some of the gray literature. Thus, more studies are needed to further investigate the diagnostic accuracy of DWI in evaluation of deep myometrial invasion.

## Conclusion

In short, even though lymphadenectomy in early (stage I) endometrial cancer is still controversial, the effect of lymphadenectomy in patients with intermediate or high risk for recurrent disease in several studies have still shown to improve survival rate. As all the poor prognostic factors for EC are interrelated, information about each factor could be equally important to clinician in decision making. So far intra-operative or post-surgical methods hold higher accuracy in detecting most of these factors. Pre-operative evaluation of these factors in early stage especially in young patients could be useful in tailoring the surgical approach thereby preserving fertility. This being said, information on myometrial tumor invasion can only be obtained pre-operatively by imaging techniques. Among them, DWI has shown better diagnostic accuracy in predicting deep myometrial invasion and also has shown to improve detection of LNM through usage of ADC mapping. Thus, through the presented study here, it is concluded that it would be worthwhile to add DWI to standard MRI protocols if pre-operative evaluation of prognostic factor is being considered in endometrial cancer patients. However, further larger prospective studies are still needed to establish the value of DWI for detecting deep myometrial invasion in patients with endometrial carcinoma.

## Competing interests

The authors declare that they have no competing interests.

## Author's contributions

SKD conceived and designed the study. XKN and JLW searched relevant article and extracted the data which was rechecked by LCZ to resolve any disagreement between the two data retrievers. WXW performed statistical analysis. SKD and AB analyzed the result and SKD wrote the paper. HFY revised the final manuscript critically for important intellectual content. All the authors read and approved the final manuscript.
